# Editorial: The immune response to therapeutic antibodies

**DOI:** 10.3389/fimmu.2025.1554297

**Published:** 2025-02-10

**Authors:** Michael G. Tovey, Arno J. Kromminga, Daniel T. Mytych

**Affiliations:** ^1^ Department of Research & Development Svar Lifescience AB, Villejuif, France; ^2^ Department of Biolytics BioNtech, Mainz, Germany; ^3^ Department of Clinical Immunology Amgen, Thousand Oaks, CA, United States

**Keywords:** therapeutic antibodies, MAPPS, monoclonal antibodies, anti-drug antibodies, neutralizing antibodies

The objective of the Research Topic of Frontiers in Immunology on the “*Immune Response to Therapeutic Antibodies*” was to provide a forum for articles that contribute to our understanding of the advances that have helped make therapeutic antibodies one of the most successful classes of therapeutic proteins, and how the field continues to develop and what are the principal challenges that limit this development. The development of an unwanted immune response including the formation of anti-drug antibodies (ADAs) remains one of the principal concerns limiting the use of therapeutic antibodies as illustrated by the recent draft guidance from the Food and Drug Administration (FDA) on how the incidence and clinical significance of ADAs should be reported on product labels. An analysis of this guidance is outlined in detail in one of the articles in the Research Topic (Swanson). The effect of ADAs can range from mere detection to a substantial effect on efficacy and safety most severe in the rare cases when the ADAs cross-react with an endogenous non-redundant counterpart. The diversity of the manuscripts received reflects the vitality of the field some 26 years after the approval of Remicade (infliximab) initially for the treatment of Crohn’s disease. These include a better understanding of the factors that predict immunogenicity risk and the outcome in patients treated with anti-tumor necrosis factor alpha antibodies (Spencer et al.; Spencer et al.). The articles published in the Research Topic demonstrate that the field continues to be innovative in terms of the techniques used to render therapeutic antibodies less immunogenic, including computational humanization of antibodies and nanobodies (Gordon et al.), and sufficiently mature to be able to compare framework shuffling and complementarity determining region (CDR) grafting for the humanization of a murine antibody (Wang et al.). Although the reduction in the immunogenicity of therapeutic antibodies has been attained primarily through sequence optimization, the introduction of chemical modifications such as PEGylation to improve serum half-life and glycosylation of the Fc domain to improve effector function can create potentially immunogenic neoepitopes that require the development of additional assays (Hagman et al.). ADAs directed against therapeutic antibodies can reduce bioavailability and alter pharmacokinetics, necessitating comprehensive immunogenicity risk assessments starting at an early stage of drug development. Given the complexity of immunogenicity, no single assay can universally predict the immune response leading to the formation of anti-drug antibodies, requiring an integrated analytical platform to comprehensively evaluate ADA against a therapeutic antibody (Ding et al.). A trend appears to be the use of a risk-centered approach involving extensive characterization and multiple assays to assess both binding and neutralizing antibodies to support the clinical development of complex multiple-domain protein therapeutics (Hagman et al.). This is illustrated by articles describing the use of a pharmacokinetic (PK) assay as the basis for the characterization of the ADA response to a T-cell engager bispecific antibody (Lotz et al.) and immunogenicity data in a non-human primate (NHP) study that informed on non-sequence, mechanism-based immunogenicity risk of a bi-functional fusion protein combining an anti-PD-1 antibody domain and a single IL-21 mutein domain on the C-terminus that translated to clinical immunogenicity risk (Kroenke et al.). The latter study showed that the cytokine domain can enhance the antibody response directed against the antibody domain, again increasing the complexity of what drives an ADA response. The importance of preclinical immunogenicity risk evaluation was illustrated by a report that showed that the ADA response to a T-cell engager administered by the subcutaneous route in patients with metastatic castration-resistant prostate cancer was markedly reduced when administered by intravenous infusion due most probably to the presence of non-tolerant T-cell epitopes within the amino acid sequence of the T-cell engager that was exposed upon subcutaneous administration (Penny et al.).

ADA production is triggered by a cascade of events initiated by antigen uptake by professional antigen-presenting cells (APCs), particularly dendritic cells (DCs). These cells process the internalized antigen and display peptide fragments as peptide–MHC-II complexes on their surface. T cells that recognize these complexes, along with receiving additional co-stimulatory signals, can trigger B-cell activation, leading to the production of ADAs (Siegel et al.). Given the pivotal role of DCs in this process, assays such as MHC-II-associated peptide proteomics (MAPPs) are frequently employed in drug development to evaluate their capacity to present drug-derived peptides (Jankowski et al.). Several articles describe *in vitro* methods to assess the immunogenicity of therapeutic antibodies based on their ability to be presented by HLA alleles to T cells (Walsh et al.) and how this can differ between geographically diverse populations and how it is crucial to predict potential adverse events and design safer biologics (Siegel et al.). The MAPPs assay has emerged as the predominant method to evaluate the immunogenic potential of engineered variants of immunogenic proteins including therapeutic antibodies (Jankowski et al.). The numerous manuscripts of high quality submitted for publication in the Research Topic of Frontiers in Immunology on the “*Immune Response to Therapeutic Antibodies”* attest to the vitality of the field and will lead to the preparation of a subsequent volume.

